# The amyloid hypothesis of Alzheimer's disease at 25 years

**DOI:** 10.15252/emmm.201606210

**Published:** 2016-03-29

**Authors:** Dennis J Selkoe, John Hardy

**Affiliations:** ^1^ Ann Romney Center for Neurologic DiseasesDepartment of NeurologyBrigham and Women's Hospital and Harvard Medical SchoolBostonMAUSA; ^2^ Reta Lila Weston Institute and Department of Molecular NeuroscienceUCL Institute of NeurologyLondonUK

**Keywords:** Aβ, Alzheimer, genetics, cell biology, treatment, Neuroscience

## Abstract

Despite continuing debate about the amyloid β‐protein (or Aβ hypothesis, new lines of evidence from laboratories and clinics worldwide support the concept that an imbalance between production and clearance of Aβ42 and related Aβ peptides is a very early, often initiating factor in Alzheimer's disease (AD). Confirmation that presenilin is the catalytic site of γ‐secretase has provided a linchpin: all dominant mutations causing early‐onset AD occur either in the substrate (amyloid precursor protein, APP) or the protease (presenilin) of the reaction that generates Aβ. Duplication of the wild‐type APP gene in Down's syndrome leads to Aβ deposits in the teens, followed by microgliosis, astrocytosis, and neurofibrillary tangles typical of AD. Apolipoprotein E4, which predisposes to AD in > 40% of cases, has been found to impair Aβ clearance from the brain. Soluble oligomers of Aβ42 isolated from AD patients' brains can decrease synapse number, inhibit long‐term potentiation, and enhance long‐term synaptic depression in rodent hippocampus, and injecting them into healthy rats impairs memory. The human oligomers also induce hyperphosphorylation of tau at AD‐relevant epitopes and cause neuritic dystrophy in cultured neurons. Crossing human APP with human tau transgenic mice enhances tau‐positive neurotoxicity. In humans, new studies show that low cerebrospinal fluid (CSF) Aβ42 and amyloid‐PET positivity precede other AD manifestations by many years. Most importantly, recent trials of three different Aβ antibodies (solanezumab, crenezumab, and aducanumab) have suggested a slowing of cognitive decline in *post hoc* analyses of mild AD subjects. Although many factors contribute to AD pathogenesis, Aβ dyshomeostasis has emerged as the most extensively validated and compelling therapeutic target.

**Glossary**
Microgliosis: early non‐specific proliferation and migration of microglial cells, macrophage‐like cells in the central nervous system, as the first response to brain damage.
Astrocytosis: final response to brain damage and injury with proliferation of astrocytes, a type of glial cell responsible for maintaining extracellular ion and neurotransmitter concentrations, modulating synapse function, and forming the blood–brain barrier.
Neurofibrillary tangles: accumulation of hyperphosphorylated tau protein, commonly found in Alzheimer's disease, that aggregates inside nerve cell bodies, also known as dystrophic neurites.
Plaque deposition: aggregates of amyloid fibrils that are deposited outside neurons in dense formations, also known as senile plaques or neuritic plaques.
FAD: familial AD caused by inherited mutations in APP and presenilin (typically early‐onset) by opposition to “sporadic” or late‐onset AD

## Introduction

Few problems in modern biomedicine have garnered as much scientific interest and public concern as has Alzheimer's disease. Virtually unknown to the general public four decades ago, AD has risen in prevalence to an estimated 40 million patients worldwide. The true number must be much higher, given the increasing recognition that the disease begins in the brain at least 2–3 decades before one first forgets the name of a grandchild or where one has parked one's car. Since molecular studies of AD began in earnest in the early 1980s, thousands of scientists and healthcare professionals have delved into all aspects of this complex, multifactorial syndrome, hoping to help patients now and prevent others from developing it in the future.

Although the progressive buildup of amyloids of diverse protein composition in various systemic organs has been known to cause devastating diseases for more than a century, the idea put forward by George Glenner (Glenner & Wong, 1984) that the particular amyloidogenic protein accumulating in AD (Aβ) could be causative has met with considerable skepticism over the ensuing years. Precisely why this idea has been so controversial is not clear (Selkoe, 2011), but the steady accrual of data from many preclinical and clinical studies has increasingly supported it. The amyloid (or Aβ) hypothesis (Beyreuther & Masters, 1991; Hardy & Allsop, 1991; Selkoe, 1991; Hardy & Higgins, 1992) has become the dominant model of AD pathogenesis and is guiding the development of potential treatments.

**Figure 1:**
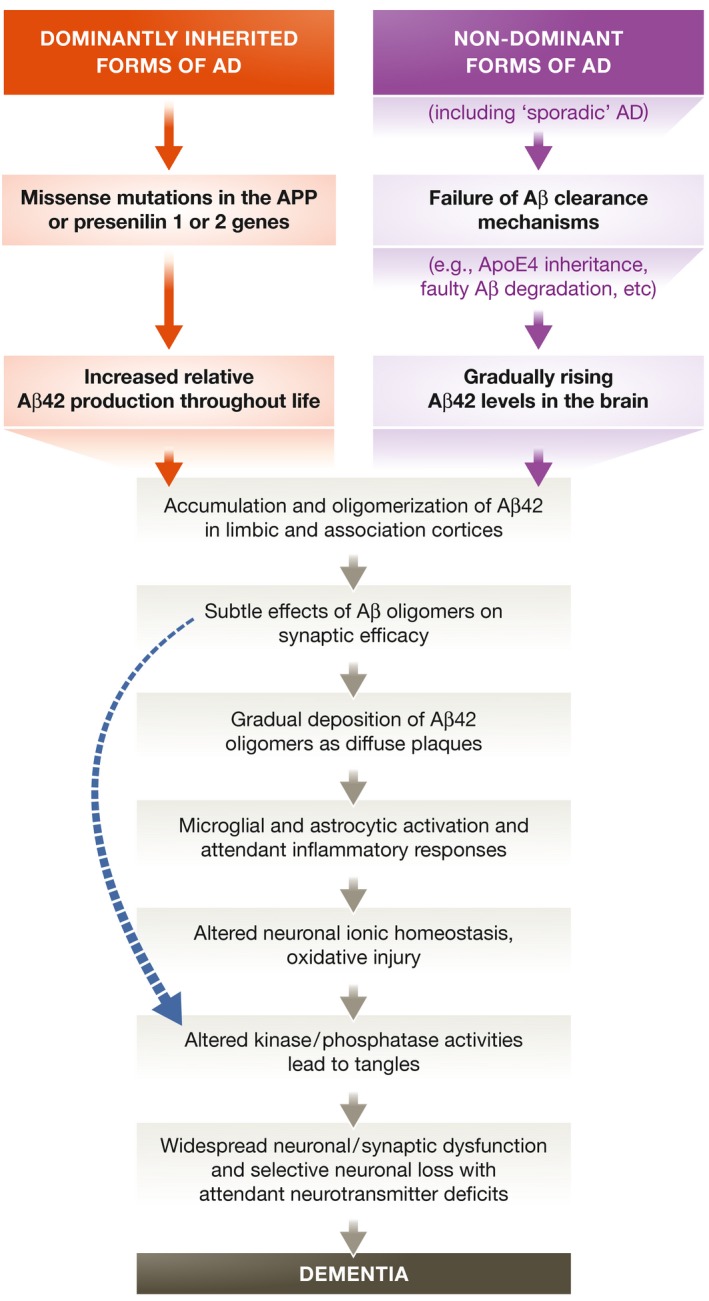
The sequence of major pathogenic events leading to AD proposed by the amyloid cascade hypothesis The curved blue arrow indicates that Aβ oligomers may directly injure the synapses and neurites of brain neurons, in addition to activating microglia and astrocytes.

We reviewed the evidence for this hypothesis (Fig 1) a dozen years ago (Hardy & Selkoe, 2002). Space precludes a full examination here of the enormous literature on Aβ since that review; a monograph on AD pathobiology contains many details (Selkoe *et al*, 2012). But in the context of continuing concern about the concept and yet the recent emergence of apparently positive clinical trial data, a critical analysis of the latest developments in laboratory and clinic is warranted and timely. We review here numerous new developments since our prior review of this hypothesis, on which ever‐increasing scientific effort is being expended. We also summarize the salient findings over three decades that undergird the amyloid hypothesis (Box 1), and we discuss several alternative concepts or concerns that have been counterposed to it (Table 1).

**Box 1: Evidence supporting a key role for Aβ dyshomeostasis in initiating AD**

All AD patients undergo progressive Aβ deposition followed by surrounding neuritic and glial cytopathology in brain regions serving memory and cognition.

Mutations within and immediately flanking the Aβ region of APP cause aggressive forms of FAD.

Humans with trisomy 21 (Down's syndrome) harbor 3 copies of APP and invariably develop neuropathologically typical AD. Those who die in their early‐to‐mid teens (from other causes) show abundant diffuse Aβ plaques without neuritic dystrophy, microgliosis, astrocytosis, and tangle formation, all of which accrue gradually in such subjects in the late teens and beyond.

Inheritance of a missense mutation in APP that decreases the production and aggregation of Aβ lifelong protects against AD and age‐related cognitive decline.

Missense mutations in presenilin 1 or 2 are the most common cause of early‐onset AD, and presenilin is the catalytic subunit of γ‐secretase. The mutations result in relative increases in the production of Aβ42/43 peptides. These hydrophobic species self‐aggregate, leading to profound Aβ deposition in mid‐life.

ApoE4 carriers were once included in typical late‐onset AD. This allele was found to markedly increase AD risk and decrease brain clearance of Aβ, leading to excess Aβ aggregation and typical downstream AD neuropathology.

Aβ42 oligomers isolated from typical (late‐onset) AD brains decrease synapse density, inhibit LTP, and enhance long‐term synaptic depression in rodent hippocampus, and their intraventricular injection impairs memory in healthy adult rats.

Human Aβ42 oligomers induce tau hyperphosphorylation at AD‐relevant epitopes and cause neuritic dystrophy in cultured rat neurons; co‐administering Aβ antibodies fully prevents this.

Aβ oligomers occur in a penumbra around many neuritic plaques. Accordingly, synapse decreases occur in a centrifugal gradient: less abnormality at longer distances from the plaque edge.

Based on many human biomarker studies, low CSF Aβ42 and positive amyloid‐PET scans precede other AD‐related changes (increased CSF tau, decreased cerebral glucose metabolism, brain atrophy, clinical dementia) by years.

Trials of 3 different Aβ monoclonal antibodies (solanezumab, crenezumab, and aducanumab) have suggested slowing of cognitive decline in *post hoc* analyses of mild (but not moderate) AD patients.

Other amyloidogenic proteins have been proven to cause progressive human organ failure, and therapeutic lowering of the amyloid or its precursor protein yields therapeutic benefits in patients.

**Table 1:** Findings that appear to undercut the amyloid hypothesis of AD and counterarguments that could explain these discrepancies

Findings	Counterarguments
Amyloid plaque burden correlates much less well with degree of cognitive impairment than do neurofibrillary tangle counts	Aβ deposits appear to be a very early and widespread event that is distant to the clinical dementia and can lead to many downstream cellular and molecular changes (e.g., microgliosis, neuritic dystrophy, tangles, etc.) that are more proximate to and causative of neuronal dysfunction
Many humans show sometimes abundant Aβ deposits at death but were not noticeably demented	Some or many of these deposits are diffuse plaques (not rich in abnormal neurites and glia); the patients were often not tested rigorously before death; and Aβ oligomer levels per plaque are much lower than in AD brains (Esparza et al, 2013), suggesting that plaques can effectively sequester oligomers in a non‐diffusible, less neurotoxic state, at least up to a point
Some human neuropathological studies suggest tangles may precede amyloid plaques	Such studies may not have searched systematically for diffuse plaques or soluble Aβ oligomers in the brain. Human genetics proves that Aβ‐elevating APP mutations lead to downstream alteration and aggregation of wild‐type tau, whereas tau mutations do not lead to Aβ deposition and amyloid‐related dementia
A hypothesis that AD is fundamentally due to loss of presenilin function has been put forward	AD‐causing presenilin mutations may indeed act through partial loss of function of this protease, but these heterozygous mutations do not produce clinically detectable loss of presenilin function (e.g., Notch phenotypes), and organismal development and function are normal until the carriers develop typical AD symptoms in mid‐life, heralded by elevated Aβ42/43 to Aβ40 ratios. Moreover, 99.9% of all AD patients have wild‐type presenilins
Numerous clinical trials of anti‐amyloid agents have not met their pre‐specified endpoints	Several of these agents had inadequate preclinical data, poor brain penetration, little human biomarker change, and/or low therapeutic indexes (e.g., tramiprosate; R‐flurbiprofen; semagacestat). Most such failed trials enrolled many patients in the late‐mild and moderate stages of AD, whereas other trials conducted in very mild or mild AD produced suggestive evidence of clinical benefit. AD trials done prior to obligatory amyloid‐PET imaging turned out to have up to ~25% of subjects that were amyloid‐negative (i.e., did not have AD)

## New insights from AD genetics and APP homeostasis

**Figure 2:**
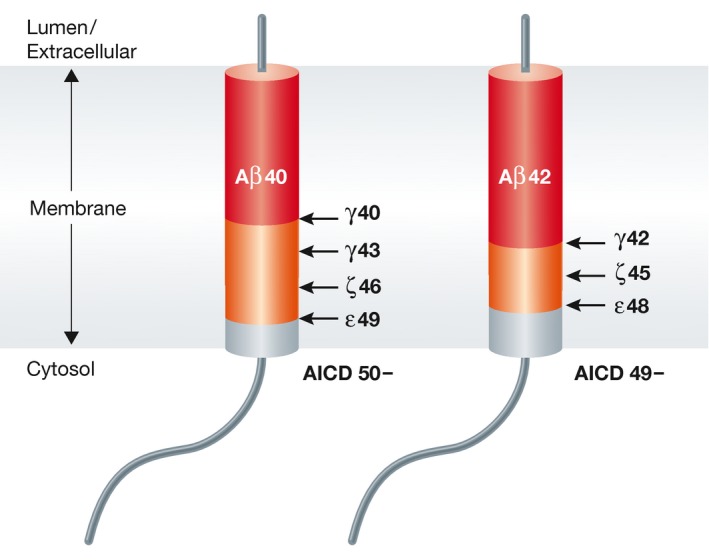
Progressive cleavages of the APP transmembrane domain by the Presenilin/γ‐secretase complex

The fact that AD‐causing mutations in APP and in presenilins 1 and 2 alter APP proteolytic processing in a way that elevates the *relative* levels of the Aβ42 or Aβ43 peptides has long been known (Scheuner *et al*, 1996; NB: Those mutations in APP that lie within the Aβ sequence increase the self‐aggregation of the resultant peptides, not their production). A key mechanistic explanation was the discovery that the presenilin genes encode the active site of the intramembrane‐cleaving γ‐secretase enzyme (De Strooper *et al*, 1998; Wolfe *et al*, 1999). Subsequent studies have begun to illuminate how presenilin mediates intramembrane proteolysis (Qi‐Takahara *et al*, 2005; Takami *et al*, 2009; Chavez‐Gutierrez *et al*, 2012; Okochi *et al*, 2013; Fernandez *et al*, 2014): an initial endopeptidase cleavage of APP near the transmembrane/cytoplasmic interface of APP (the ε‐cleavage) is followed by multiple carboxypeptidase cleavages that each sequentially removes 3 or 4 C‐terminal amino acids (i.e., approximately one turn of the intramembrane helix) (Fig 2). This process yields two product lines that start with either the Aβ48/49 or the Aβ49/50 ε‐cleavage. Although the precise molecular effects of different presenilin mutations differ somewhat, in all cases the mutations appear to decrease this C‐ to N‐terminal cleavage “processivity” and thus increase the relative production of longer (more hydrophobic and self‐aggregating) Aβ peptides. This elegant model provides a biochemical explanation for earlier work showing that pathogenic presenilin mutations often increase the Aβ42/Aβ40 ratio in humans. γ‐Secretase reactions conducted directly in presenilin‐mutant AD brain tissue showed that all presenilin mutations studied decreased this carboxypeptidase‐like activity, and assays in a few “sporadic” AD brains suggested that a similar decrease in processivity might occur in some non‐presenilin‐mutant cases (Szaruga *et al*, 2015). Aβ42, Aβ43, and longer Aβ peptides are highly self‐aggregating, whereas Aβ40 may actually be anti‐amyloidogenic (Kim *et al*, 2007).

One group has emphasized that the aforementioned mechanism represents a loss of function of presenilin and have proposed that the neural phenotype of AD patients is fundamentally due to a loss of presenilin function, independent of effects on Aβ production (Shen & Kelleher, 2007; Xia *et al*, 2015). They have studied presenilin‐1 mutations that generally lower Aβ and hardly raise relative Aβ42 levels, but this work may overlook an elevation of the Aβ43 and other longer species, which are highly amyloidogenic (Saito *et al*, 2011). Although AD‐causing presenilin mutations can indeed be interpreted as partial loss of function from a genetics perspective, pinpointing the function of presenilin as an aspartyl endopeptidase allows one instead to speak in biochemical terms of a functional shift of the principal proteolytic cleavages to more C‐terminal bonds in the substrate (Kretner *et al*, 2016). Humans with pathogenic presenilin mutations are heterozygotes and experience no loss of function of Notch cleavage; rather, they have accelerated Aβ42 and Aβ43 accumulation that long precedes their AD‐typical memory syndrome. Most importantly, > 99% of all AD patients (including all other forms of familial disease) express wild‐type presenilin, so loss of presenilin function cannot be a general mechanism of AD pathogenesis.

The original formulation of the amyloid hypothesis was based in part on the discovery that the APP gene is on chromosome 21, implying that individuals with Down's syndrome develop typical Alzheimer neuropathology because they produce too much Aβ lifelong. This supposition has been substantiated by the identification of humans with different segmental microduplications of sub‐regions of chromosome 21. Rare individuals with translocation Down's syndrome involving only the distal part of chromosome 21 telomeric to the APP gene have Down's features but do not get AD (Prasher *et al*, 1998). Conversely, those rare individuals who have the APP gene micro‐duplicated but not the rest of the chromosome do not have Down's syndrome but get AD, typically in their mid‐50s (Rovelet‐Lecrux *et al*, 2006). These findings show conclusively that lifelong overexpression of wild‐type APP causes AD. Even more remarkable has been the identification of an APP missense mutation (A673T) at the second amino acid of the Aβ region that results in a lifelong decrease in APP cleavage by β‐secretase (Jonsson *et al*, 2012). Moreover, this benefit may be compounded, because the mutant Aβ peptide that is generated has altered aggregation properties (Benilova *et al*, 2014; Maloney *et al*, 2014; Zheng *et al*, 2015). A673T carriers have a lower risk of clinical AD and even of age‐related cognitive decline without clinical AD (Jonsson *et al*, 2012), and they may not show plaque deposition at age 100 (Kero *et al*, 2013). The reduced amyloid deposition resulting from this AD‐protective mutation strongly supports the amyloid hypothesis.

## Improved modeling of the amyloid hypothesis in rodent and cellular systems

Concern has been expressed about the limitations of available rodent and cellular models of β‐amyloid pathogenicity (Table 2). Early APP mouse models (e.g. Games *et al*, 1995; Hsiao *et al*, 1996) suffered from reliance on high transgene expression to drive plaque deposition and from a lack of tangle cytopathology and neuronal death. Crossing FAD‐mutant APP mice with mutant *MAPT* (tau) transgenic (tg) mice succeeded in augmenting tau pathology and suggested that tangle‐like changes occur downstream of Aβ accumulation, but this involved transgene overexpression and multiple AD mutations (Lewis *et al*, 2001). Recently, mice with gradual Aβ plaque accrual have been developed by the judicious use of selective knockin of human mutations into endogenous mouse APP without overexpression (Saito *et al*, 2014). Moreover, stem cell‐derived human neurons cultured from skin biopsies of FAD subjects have been used to show first Aβ accumulation and then tau alteration in the absence of overexpression (Shi *et al*, 2012; Choi *et al*, 2014; Muratore *et al*, 2014; Moore *et al*, 2015) suggesting that the lack of tangle formation in early mouse models was related to the absence of human tau. This progress means we are now able to model a substantial part of the amyloid cascade in culture. In both cellular and mouse models, extensive data now suggest that the neurotoxicity of Aβ is in considerable part dependent on expression of human tau (Rapoport *et al*, 2002; Jin *et al*, 2011; Roberson *et al*, 2011).

**Table 2:** Toward a more complete modeling of the pathogenesis of AD amyloid

Year	System	Achievement	Critique	References
1995	APP transgenic mouse	Plaque Pathology	Overexpression, no downstream pathology	Games *et al* (1995)
2000	MAPT mutant transgenic mouse	Tangle Pathology	Overexpression: no plaque pathology	Lewis *et al* (2000)
2001	APP X MAPT transgenic mice	Plaque and tangle pathology	Overexpression of both transgenes: artificiality of two mutations	Lewis *et al* (2001)
2012	Down's syndrome derived stem cell neurons	Diffuse plaque pathology: evidence for pre‐tangles	Not full pathology	Shi *et al* (2012)
2014	Complex APP mutation knockin into mouse genome	Plaque pathology without overexpression	Artificiality of multiple mutations: no downstream pathology	Saito *et al* (2014)
2014	Overexpression of APP mutations in human neuronal lines in gel system	Convincing plaque pathology and also tangle pathology	Overexpression	Choi *et al* (2014)
2015	APP and PSEN mutant stem cell lines	Diffuse plaque pathology and tau pathology		Moore *et al* (2015)

## Cell biology of new AD risk genes

Although the importance of ApoE4 as the major risk factor for AD was discovered in 1993 (Corder *et al*, 1993), it is only since the advent of genomewide association studies and, more recently, exome and genome sequencing that other risk loci for late‐onset disease have been discovered. Whereas the recently described loci are usually much weaker in effect (Lambert *et al*, 2013) or much rarer (Guerreiro *et al*, 2013; Jonsson *et al*, 2013) than ApoE4, they have helped delineate additional biological processes in AD pathogenesis. Three types of processes have emerged as especially important: cholesterol/sterol metabolism; inflammation and the brain's innate immune system; and endosomal vesicle recycling (Jones *et al*, 2010).

### Apolipoprotein E and other components of cholesterol/sterol metabolism

A role for cholesterol in AD has long been suspected, based on the genetic implication of ApoE in the disease as well as the contrasting effects of cholesterol loading or depletion on amyloid pathology in APP tg mice (Refolo *et al*, 2000, 2001). Work in APP mice expressing different human ApoE alleles has shown that a major pathogenic influence of ApoE involves differential isoform effects on the clearance of Aβ (Castellano *et al* 2011: discussed below). The ABCA7 lipid transporter has also been identified as a genetic locus for the disease (Hollingworth *et al*, 2011), and loss‐of‐function mutations increase AD risk about threefold (Steinberg *et al*, 2015). ABCA7 is expressed in neurons, microglia, and peripheral macrophages, and it normally promotes the efflux of lipids from cells to apolipoproteins and also regulates phagocytosis. Crossing ABCA7 knockout mice to mutant hAPP mice caused a doubling of insoluble Aβ levels and amyloid plaques without changing APP processing, suggesting that like ApoE, ABCA7 is involved in Aβ clearance (Kim *et al*, 2013). However, the biochemical details through which both ApoE and ABCA7 influence the development of Aβ pathology need to be pinpointed.

### The innate immune system in Alzheimer's disease

Neuropathologists have long suggested that the brain's innate immune system, including the microglial response to plaque formation, was an important factor in AD pathogenesis. For example, the early observation of multiple elements of the classical complement cascade in and around neuritic plaques (McGeer *et al*, 1989) was prescient. In the last few years, genetic variability in that system has emerged as a compelling determinant of AD risk, implicating many components of innate immunity and the complement cascade as risk factors in the disease (Jones *et al*, 2010). Three such risk genes have been investigated in some detail: Complement Receptor 1 (CR1; Lambert *et al*, 2009), CD33 (Bertram *et al*, 2008), and TREM2, and all three appear to be involved either directly or indirectly in the response of microglia to Aβ deposition. Blockade of CR1 inhibits microglial activation and potentiates microglial phagocytosis (Crehan *et al*, 2013). Inactivation of CD33 in primary microglia also potentiates microglial uptake of Aβ (Griciuc *et al*, 2013), and TREM2 is responsible for sustaining microglial phagocytosis of Aβ (Wang *et al*, 2015). Thus, all three genetically implicated microglial proteins may be involved in helping to maintain the AD microglial phenotype of phagocytosing Aβ deposits. Accordingly, these 3 genes undergo increased expression during plaque development (Griciuc *et al* 2013, Wang *et al*, 2015; Matarin *et al*, 2015) and CSF TREM2 levels go up as plaque load increases, suggesting it may be a useful biomarker (Suárez‐Calvet *et al*, 2016).

TREM2 is emerging as a key molecular determinant of the CNS response to Aβ accumulation (Forabosco *et al*, 2013; Zhang *et al*, 2013; Matarin *et al*, 2015). However, the biology of TREM2, a Type 1 single‐transmembrane receptor which is principally but not exclusively expressed in microglia and undergoes ADAM/γ‐secretase processing (Wunderlich *et al*, 2013; Kleinberger *et al*, 2014), is incompletely understood [reviewed in (Lue *et al*, 2015)]. The most studied mutation, R47H, may increase the risk of AD to the same extent that ApoE4 does although it is much rarer (Guerreiro *et al*, 2013; Jonsson *et al*, 2013). The upregulation of TREM2 in a subset of microglia in amyloid plaques of hAPP tg mice (e.g., Guerreiro *et al*, 2013) suggests that the known function of TREM2 in phagocytosis is compromised during plaque development. A current hypothesis is that R47H and other AD‐associated TREM2 mutations confer loss of function in microglia. Deleting one TREM2 allele in hAPP tg mice significantly decreased the number of microglia associated with Aβ deposits (Ulrich *et al*, 2014). Conversely, TREM2 overexpression in hAPP tg mice decreased amyloid plaque burden, neuroinflammation, synapse loss, and spatial memory deficits (Jiang *et al*, 2014). And TREM2 mutations can alter its transport to the cell surface and shedding, associated with impaired phagocytic function (Kleinberger *et al*, 2014). The latter work has led to evidence that levels of the shed ectodomain in extracellular fluid and CSF are lower in AD cases associated with TREM2 mutations.

### Endosomal vesicle recycling in Alzheimer's disease

The final set of recently identified loci for late‐onset AD map to processes regulating endosomal vesicle recycling (Jones *et al*, 2010). This category includes SORL1, BIN1, and PICALM (Rogaeva *et al*, 2007; Lambert *et al*, 2013; Zhao *et al*, 2015). SORL1 had previously been shown to be directly involved in the processing of APP (Andersen *et al*, 2005), and work in human stem cell‐derived neurons carrying the SORL1 risk haplotype confirmed this association (Young *et al*, 2015). Likewise, PICALM appears to be involved directly in endosomal APP processing (Kanatsu *et al*, 2014). In addition, PICALM has been implicated in the transport of brain Aβ across the blood–brain barrier: induced pluripotent stem cell (iPSC)‐derived human endothelial cells carrying an AD‐protective allele exhibited higher PICALM levels and enhanced Aβ clearance (Zhao *et al*, 2015).

In summary, mechanistic studies linking several of the recently identified risk genes for late‐onset (previously “sporadic”) AD to aspects of Aβ homeostasis provide new support for the amyloid hypothesis as a driving factor in AD pathogenesis. They also suggest new avenues for therapeutic intervention, such as intervening in brain cholesterol metabolism and modulating the response of the innate immune system to amyloid deposition.

## Recent findings help resolve controversies about the role of Aβ

### Connecting plaques and tangles: Aβ can drive tau alteration

The temporal sequence of the two canonical lesions Alois Alzheimer noted in his 1906 index case has been debated ever since. An elegant histopathological staging system created by Braak and Braak (1991) is now widely used to establish the severity of AD neuropathology. This scale principally described the progression of AD‐type cytoskeletal changes, that is, neurofibrillary tangles and dystrophic neurites, in unrelated humans of increasing age (it could not yet include assays for accrual of oligomeric forms of Aβ). The detection of modest amounts of neurofibrillary change in limbic regions of young or middle‐aged individuals dying of other causes does not imply that such individuals would necessarily have developed AD had they lived longer. Instead, human genetic and biomarker studies have provided the answer to the sequence of Aβ and tau accumulation in AD. Inherited mutations in APP and presenilin (i.e., in the substrate and the protease for Aβ generation) cause early‐onset Aβ deposition (Lemere *et al*, 1996a,b; Bateman *et al*, 2012) followed by accumulation of tangles/neurites containing filaments of wild‐type tau, so amyloid can clearly precede tangles in humans. In contrast, mutations in the tau gene lead to a form of frontotemporal dementia without subsequent accrual of Aβ. Thus, Aβ accumulation can lead to progressive tau deposition, but the converse has not been clearly demonstrated in humans.

Laboratory studies support this sequence. Crossing hAPP tg mice with hTau tg mice significantly enhances tau deposition without changing Aβ deposition (Lewis *et al*, 2001). Crossing an APP tg mouse to a tau knockout mouse leads to substantially less behavioral deficits in the offspring than when tau is expressed (Roberson *et al*, 2011). Treating normal rat neurons in culture with soluble Aβ oligomers isolated from AD cortex causes neuritic dystrophy and AD‐type tau hyperphosphorylation, but no dystrophy ensues if tau is first knocked down (Jin *et al*, 2011). Several similar studies suggest that Aβ—particularly soluble oligomers of Aβ42 (Shankar *et al*, 2008)—can trigger AD‐type tau alterations, supporting the sequence that human genetics has indicated. The expression of human tau seems to be “permissive”, enabling certain downstream neuronal consequences of progressive Aβ accrual to occur (Maruyama *et al*, 2013).

### How ApoE4 promotes AD: chronically decreased Aß clearance

Humans expressing the ApoE4 protein develop more plaque and vascular β‐amyloid deposits than those expressing only ApoE3 (Rebeck *et al*, 1993), and this has been confirmed in genetically engineered mice (Holtzman *et al*, 2000). A detailed quantitative study of Aβ homeostasis using *in vivo* microdialysis in hAPP × hApoE crossed mice has shown that Aβ clearance (but not Aβ production) is decreased by ApoE4 > E3 > E2, closely paralleling the degree of Aβ deposition in such mice (Castellano *et al*, 2011). The decrease in clearance of soluble Aβ was observed in young mice well before any amyloid deposition. The results strongly suggest that ApoE contributes to AD risk at least in part by differentially regulating soluble Aβ clearance, emphasizing Aβ clearance pathways as a major therapeutic target. In accord, one of the numerous risk genes for late‐onset AD is PICALM, and AD‐promoting alleles or knockdown of this gene has been shown to decrease Aβ clearance across the brain endothelium (Zhao *et al* 2015). As a potentially related effect on Aβ homeostasis, the three ApoE isoforms have also been shown to bind Aβ differentially and modulate its fibrillogenesis (Ma *et al*, 1994; Wisniewski *et al*, 1994; Evans *et al*, 1995). Other potential mechanisms have been suggested, including an adverse effect of ApoE4 on the processing of tau in neurons (Andrews‐Zwilling *et al*, 2010; Huang & Mahley, 2014).

### Synaptic loss, Aβ, and amyloid plaques

Decreased synapse number has long been recognized as perhaps the strongest quantitative neuropathological correlate of dementia in AD. Numerous laboratory studies in the past decade have shown that Aβ oligomers impair both synaptic function (e.g., long‐term potentiation) and synaptic structure (e.g., dendritic spines). Of particular, disease relevance is evidence that soluble oligomers (but not monomers) of Aβ42 isolated directly from AD cortex can dose‐dependently decrease synaptic function and number and can impair the memory of a learned behavior in healthy adult rats (Shankar *et al*, 2008). Amyloid plaque cores isolated from the same AD brains and washed extensively *in vitro* do not impair LTP, but the diffusible Aβ42 oligomers that can subsequently be released from them with harsh denaturants do so (Shankar *et al*, 2008). The latter findings fit nicely with evidence in hAPP tg mice that plaques *in situ* have a penumbra of soluble Aβ oligomers in which synaptic density is low; synapse number rises toward normal the farther one measures from the edge of the plaque core (Koffie *et al*, 2009).

The intimate association of diffusible oligomers with fibrillar plaques that such studies imply has been elegantly supported by quantifying Aβ oligomers with a selective ELISA in postmortem brain tissue of subjects who were either clinically normal (Clinical Dementia Rating of 0) or mildly demented (CDR of 1) shortly before death. These brains were selected to have similar plaque densities, but the oligomer‐specific ELISA revealed that the non‐demented plaque‐rich subjects had much lower oligomer‐to‐plaque ratios than the mildly demented plaque‐rich patients (Esparza *et al*, 2013). Indeed, this ratio completely distinguished (without overlap) the “high‐pathology control” brains from the AD brains. This striking result addresses an oft‐cited “Achilles heel” of the amyloid hypothesis: apparently normal people who have abundant plaques may actually have low plaque‐associated oligomer levels (Table 1). We have hypothesized that plaques can sequester soluble oligomers until they slowly reach a physical limit, after which excess oligomers can diffuse onto surrounding synaptic membranes and other hydrophobic cell surfaces (Hong *et al*, 2014).

### A heterogeneity of Aβ species in AD brain

While Aβ1–42 peptides appear to be the earliest form to accumulate in the brain and their free levels in CSF drop long before clinical symptoms [e.g., Bateman *et al* (2012)], this initial species can be modified over time into a complex array of truncated, isomerized, and/or phosphorylated peptides. One well‐studied variant that is highly amyloidogenic (Nussbaum *et al*, 2012) is the “p3E” Aβ peptide that is truncated over time of Asp1 and Ala2 and then cyclized at Glu3 (Mori *et al*, 1992). DeMattos *et al* (2012) have shown in hAPP transgenic mice that this variant accumulates rather late and in small amounts, but targeting it with a specific antibody promotes a kind of “bystander clearance” by microglia of also earlier deposited Aβ species, making p3E an attractive target for Aβ immunotherapy despite its low abundance. At the opposite end of Aβ, the variant Aβ43 is highly prone to aggregation (Saito *et al*, 2011), and it is unclear how long soluble oligomers of this peptide can be present as such in the brain before they deposit as insoluble amyloid plaques. Given the complexity of Aβ species in humans, a worthy goal for future clinical research is to routinely quantify all Aβ peptides in plasma or CSF of pre‐symptomatic and symptomatic AD subjects.

Recent studies have uncovered further heterogeneity of both Aβ and other proteolytic products of APP processing. One example is the detection in certain cell lines expressing pathogenic mutant APP, of Aβ monomers and dimers as well as N‐terminally extended Aβ monomers that begin some ~35–40 resides before the Asp1 of Aβ (Welzel *et al*, 2014). Levels of these extended monomers rise dramatically when β‐secretase inhibitors are applied to the cells, indicating that they arise from an alternative protease(s) which cleaves APP upstream of the Met‐Asp bond. The N‐terminally extended monomers inhibit LTP, presumably because they contain the Aβ region but in a misfolded form that can bind to synaptic membranes (Welzel *et al*, 2014). A second and distinct set of APP fragments arises from a novel η (“eta”)‐secretase processing pathway, involving cleavage by a matrix metalloproteinase 92 amino acids upstream of the Met‐Asp bond (Willem *et al*, 2015). The resultant η‐CTFs can then be processed by α‐ or β‐secretase to generate Aη‐α and Aη‐β peptides. Again, β‐secretase inhibitors elevate these alternative APP products. Single‐cell calcium imaging shows that neuronal activity is attenuated by Aη‐α, suggesting a physiological function of this new pathway but not yet implicating it in AD (no η‐derived fragments containing the intact Aβ region were described; Willem *et al*, 2015).

## Is AD in humans a prion‐like disease of “pathogenic spread”?

In the last decade, the neurodegenerative field has become increasingly intrigued by the hypothesis that the progression of the cytopathological lesions in AD, Parkinson's disease, Frontotemporal dementia, and other age‐related diseases involves a physical spread of the specific offending proteins from neuron to neuron. Initially, this hypothesis was based in considerable part on three observations. First, the Braak staging of tangles (in AD) and Lewy lesions (in Parkinson's disease) in postmortem brains from humans of increasing age was interpreted as a physical spread of the responsible protein aggregates (tau and α‐synuclein, respectively) from one brain region to the next. Second, the development over many years of some Lewy bodies in fetal neurons implanted into the striata of a few advanced Parkinson patients was interpreted as a physical spread of a pathogenic form of α‐synuclein from the diseased neurons of the host into the healthy neurons of the implant. Third, experimental studies in rodents suggested that the extracellular injection of fibrils of tau or α‐synuclein could induce still‐healthy neurons to form the respective intracellular lesions, suggesting a physical spread as well as a “prion‐like” templating of the normal protein by the abnormal (misfolded) protein.

These findings provide circumstantial evidence for the spread of misfolded proteins from neuron to neuron, although the details of the cell biological mechanisms remain unclear. The extent to which the Braak staging in AD (as well as in Parkinson's disease and other neurodegenerative diseases) represents a selective temporal vulnerability of neurons in different brain regions rather than an actual physical spread has not been elucidated. It remains a challenge to distinguish cell autonomous from non‐cell autonomous mechanisms of protein aggregation *in vivo*, particularly in humans with pathogenic mutations in tau or α‐synuclein, where 50% of the protein in all neurons is mutant and could aggregate without the need for inter‐neuronal spread. Clearly, these are important unresolved issues: understanding the biological mechanisms for the spread of cytopathology could offer new therapeutic targets and may underlie the observed beneficial effects of administered antibodies on lesion clearance.

A separate question from that of neuron‐to‐neuron spread is whether the misfolded Aβ and tau aggregates in AD brain could be true “proteinaceous infectious particles” and thus transmissible between humans. Attempts by Gadjusek and colleagues more than 30 years ago to transmit AD to lower primates by inoculation of AD brain extracts were deemed unsuccessful (Brown *et al*, 1994). However, Ridley *et al* (2006) reported that inoculation of marmosets with AD brain extracts induced modest cerebral β‐amyloidosis: Aβ‐immunoreactive deposits were detected in 16 of 18 animals aged < 10 years and 8 of 9 aged > 10 years, whereas spontaneous cerebral amyloid deposition was found in 0 of 11 non‐injected marmosets < 10 years and 5 of 29 > 10 years (Ridley *et al*, 2006). Neurofibrillary tangles were not detected in any animals, so it seems that inoculation with AD brain extracts accelerated Aβ (but not tau) deposition in these primates. This acceleration is entirely consistent with the long‐standing concept of the seeded polymerization of Aβ in kinetic models of β‐amyloid formation (Jarrett & Lansbury, 1993).

Recently, four patients dying between ages 36 and 51 of iatrogenic Creutzfeld–Jakob disease after childhood treatment for short stature with prion‐contaminated cadaveric pituitary extracts were reported to also have substantial Aβ‐immunoreactive plaques and microvessels in their brains (Jaunmuktane *et al*, 2015). Since pituitary glands in AD subjects can have Aβ deposits, these cases were interpreted as providing evidence of human‐to‐human transmission of Aβ seeds. No tangles or AD‐type neuritic/microglia‐rich plaques were described, so this was not transmission of AD *per se*, consistent with an earlier negative study (Irwin *et al*, 2013). Locating some of the original pituitary extracts used for inoculation and showing that they had abnormal Aβ forms will be necessary to prove that these cases represent definite human transmission of Aβ seeds. At present, there are no clear clinical concerns arising from this unusual iatrogenic event as regards AD risk in the general population.

## Biomarkers: approaching the natural history of AD in human subjects

For many years, reaching a correct understanding of the pathogenic sequence in AD patients was hampered by the difficulty of detecting this sequence directly in living humans. The problem has been substantially lessened by three developments: (i) robust assays to quantify soluble Aβ monomers and tau in CSF (Vigo‐Pelfrey *et al*, 1995); (ii) imaging fibrillar amyloid burden (but not yet soluble Aβ oligomers) by PET scanning, initially with the thioflavin T derivative, Pittsburgh compound B (Klunk *et al*, 2004); and (iii) the ability to analyze APP metabolism in healthy and diseased humans by quantifying heavy isotope‐labeled Aβ peptides by mass spectrometry in fresh CSF collected continuously through a lumbar intrathecal catheter (Bateman *et al*, 2006; Mawuenyega *et al*, 2010).

### *In vivo* APP labeling

The use of ^13^C (heavy) leucine infusions to label all newly synthesized proteins, including APP, in pre‐symptomatic subjects with presenilin mutations and their non‐carrier siblings confirmed the extensive data in cultures and mouse models that these AD‐causing mutations increase relative Aβ42 production (Potter *et al*, 2013). Further, during the period of amyloid deposition, the Aβ42 monomer declines in CSF in a manner that suggests it becomes bound to developing plaques (Blennow *et al*, 2015). And the use of the *in vivo* labeling approach in ApoE3 vs. E4 carriers showed that E4 subjects had lower rates of Aβ monomer clearance [see (Castellano *et al*, 2011)]. Together, these human data support the conclusions that presenilin mutation carriers produce relatively more Aβ42 and that E4 carriers clear it less efficiently.

### Amyloid imaging and CSF biomarkers

Numerous families carrying *APP*,* PSEN1*, or *PSEN2* mutations have been studied collectively to determine the time course of fluid biomarker, neuroimaging, and clinical changes prior to the expected onset of AD symptoms, which is based on the age of symptom onset in a parent with the same mutation. Initial analyses of a familial AD cohort [the Dominantly Inherited Alzheimer Network (DIAN)] suggest that Aß42 levels in CSF may first be somewhat elevated (vs. normal) and then begin to decline as early as 25 years before expected symptom onset (Bateman *et al*, 2012). This is followed by the appearance of fibrillar amyloid deposits in the brain (as detected by PiB‐PET), increased levels of tau in CSF, and progressive brain atrophy roughly 15 years before expected symptom onset (Bateman *et al*, 2012). Neuronal hypometabolism and subtly impaired episodic memory seem to begin some 10 years or so before expected symptoms (Bateman *et al*, 2012). If this time course is generally similar to that of “sporadic” AD, and the AIBL study (Villemagne *et al*, 2013) suggests that it is, then Aβ deposition may begin up to two decades or more before clinically noticeable cognitive decline. A key lesson which emerges from such dynamic analyses of pre‐symptomatic AD is that therapeutic interventions directed only at the mild‐to‐moderate clinical stage may be too late to ameliorate progression.

**Figure 3:**
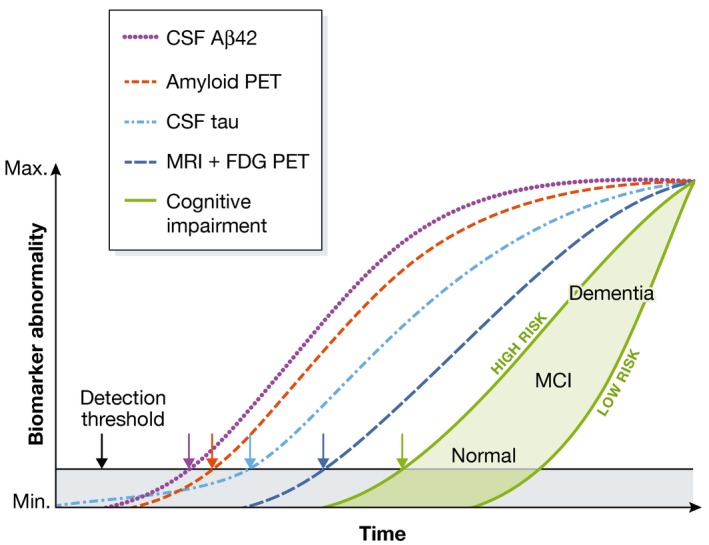
A hypothetical temporal model integrating Alzheimer's disease biomarkers The threshold for the first detection of biomarkers associated with pathophysiological changes is denoted by the black horizontal line. The gray area denotes the zone in which abnormal pathophysiological changes lie below this biomarker detection threshold. In this model, the occurrence of tau pathology can precede Aβ deposition in time, but only early on at a sub‐threshold biomarker detection level. Aβ deposition occurs independently and rises above the biomarker detection threshold (purple and red arrows). This induces acceleration of tauopathy, and CSF tau then rises above the detection threshold (light blue arrow). Later still, changes in FDG PET and MRI (dark blue arrow) rise above the detection threshold. Finally, cognitive impairment becomes evident (green arrow), with a wide range of cognitive responses that depend on the individual's risk profile (light green‐filled area). Note that while CSF Aβ42 alteration is plotted as a biomarker (purple), this represents a decrease in CSF Aβ42 levels and is a surrogate for an increase in parenchymal Aβ42 and changes in other Aβ peptides in the brain tissue. Aβ, amyloid β‐protein; FDG, fluorodeoxyglucose; MCI, mild cognitive impairment. (Adapted from Fig 6 of Jack *et al*, 2013.)

Overall, brain imaging and CSF biomarker studies in humans suggest that the sequence of AD pathogenic steps currently measurable *in vivo* broadly follows the schema proposed by Jack and colleagues (Jack & Holtzman, 2013; Jack *et al*, 2013; Fig 3). These data are consistent with early studies of AD neuropathology in Down's syndrome, which documented an initial accumulation of diffuse Aβ deposits that precedes microglial and astrocytic activation, tangle formation, and neurodegeneration (e.g., Lemere *et al*, 1996a,b; Mann *et al*, 1992). The recent development of imaging agents for tangles (Chien *et al*, 2013; Liang *et al*, 2014) will help define the time course of accrual of the two major lesions, although tangles are somewhat non‐specific in that they occur increasingly with “normal” aging and in several neurodegenerative processes besides AD.

## Recent progress in AD clinical trials

None of the Aβ‐targeted phase 3 clinical trials in Alzheimer's disease has shown statistically significant benefit on its pre‐specified clinical endpoints. Several of these trials, however, were misdesigned in terms of patient selection, choice of agent, target engagement, and/or dose, or they had to be halted because of side effects that may not have been target‐related (De Strooper, 2014; Karran & Hardy, 2014).

### Semagacestat was neither an effective nor safe γ‐secretase inhibitor

Inhibiting the β‐ or γ‐secretases is an attractive goal, but the recognition that they have many substrates besides APP makes selectivity an enormous challenge. The discovery that Aβ is normally secreted by cells throughout life (Haass *et al*, 1992) led to widespread compound screening on cultured cells, and most Aβ‐lowering “hits” that emerged inhibited γ‐secretase. The only such compound to reach Phase 3 testing was semagacestat, but the trial was terminated after ~12 months of dosing due to adverse events (Doody *et al*, 2013). This may be explained by its low therapeutic index: the IC_50_ for Notch cleavage was only twofold to threefold higher (or less) than that for APP cleavage. Direct proof that semagacestat was an effective Aβ‐lowering agent in humans was not obtained, and this trial should not have led to the curtailment of research to develop safer inhibitors of γ‐secretase with better substrate selectivity (De Strooper, 2014). Another agent, avagacestat, had a better therapeutic index but still not good enough to avoid certain side effects and be advanced to Phase 3 trials (Coric *et al*, 2012).

The fundamental catalytic mechanism of this first‐in‐class intramembrane aspartyl protease is incompletely understood, although substantial progress should ensue from biochemical studies that take advantage of the atomic structure of the whole γ‐secretase complex that has recently been solved (Bai *et al*, 2015). While further research on selective inhibition of the protease is needed, the field now favors modulators of γ‐secretase that shift the peptide bond cleavage 3–4 residues N‐terminal to the Aβ42 site without blocking proteolysis. Different chemical classes of such γ‐modulators are being developed; whether they can achieve brain penetration and potency to levels needed to lower Aβ chronically remains untested.

### Solanezumab: a probable signal in mild AD

Since active and passive immunotherapy to lower amyloid was first conceptualized (Schenk *et al*, 1999; Bard *et al*, 2000), antibody trials have taken the lead among putative disease‐modifying therapeutics for AD. The antibody most advanced time‐wise in current human testing is solanezumab, which targets the mid‐region of Aβ and binds principally to soluble monomers and perhaps low‐n oligomers but not to plaques. Two large Phase 3 trials in mild and moderate AD patients failed to achieve their clinical endpoints. Pre‐specified, *post hoc* analyses of the combined mild subjects of the trials showed a statistically significant ~34% slowing of cognitive decline vs. placebo over 18 months [see tables 3 and 4 in Doody *et al*, 2014]. The moderate AD patients in the same trials showed no benefit, proving the widely held assumption that anti‐Aβ agents should be started in mild AD or even earlier. The results in the mild subjects suggested a small but statistically significant cognitive benefit of this agent, leading to a third Phase 3 study in only mild subjects that is underway. It is of interest that another antibody, crenezumab, produced similar signs of modest slowing of cognitive decline in mild AD patients in a Phase 2 trial (Cummings *et al*, 2014).

### Aducanumab: a big signal in a small proof‐of‐concept trial

The strongest hint to date of the potential clinical and biomarker benefits of an amyloid‐targeting agent came recently in a Phase 1b trial of a human monoclonal antibody (BIIB‐037 or aducanumab) that emerged from a large screen of B‐cell clones obtained from healthy aged people. All 165 trial subjects underwent PET amyloid imaging at entry to confirm the clinical diagnosis; this had not been done in the completed trials reviewed above, where up to 30% of subjects were later found to lack amyloid. Another difference of the small aducanumab trail is that it was conducted in one country (United States), so all cognitive evaluations were performed in one common language, probably reducing inter‐subject variability in scoring. Three IV doses (1, 3 or 10 mg/kg/mo) were initially compared to placebo after 6 and 12 months. The 3 and 10 mg dose reduced PET amyloid levels at 6 months and more so at 12 months, with the 10 mg dose causing a decline to near the level required for trial entry (Sevigny *et al*, 2015). This dose‐dependent evidence of target engagement and biomarker movement was accompanied by significantly less decline (vs. placebo) in two tests, the Mini‐Mental State Exam and the Clinical Dementia Rating—Sum of Boxes. In the 10 mg dose group, these scores were almost stable from 6 to 12 months. The only meaningful adverse event was transient ARIA‐E (amyloid‐related imaging abnormality—edema) in ~20% of the subjects receiving aducanumab. As in some prior Aβ antibody trials, ARIA‐E occurred mostly in ApoE4^+^ patients, was dose‐dependent, and produced no symptoms in 65% of these cases. Both the careful design of this small study and the nature of the human antibody, which apparently binds plaques and oligomers but not monomers, may have contributed to the positive clinical and biomarker outcomes. Aducanumab entered the necessary Phase 3 studies in 2015.

### The advent of “secondary prevention” trials

The failures of some anti‐amyloid agents that appeared to engage their targets but did not achieve clinical endpoints in mild‐to‐moderate AD patients have moved the field to attempt pre‐symptomatic or “secondary prevention” trials in subjects shown by PET amyloid imaging and/or CSF Aβ42/tau assays to be at high risk for developing AD dementia. Such prevention trials are now being conducted, respectively, in the world's largest kindred carrying a presenilin‐1 mutation (the API study in rural Colombia (Ayutyanont *et al*, 2014)) or in many smaller kindreds carrying presenilin‐1 or presenilin‐2 or APP mutations (the DIAN trials). Importantly, the first prevention trial in largely pre‐symptomatic humans at risk of late‐onset AD (ages 65–85) based on abnormal PET amyloid scans is now underway in > 60 centers in the United States, Canada and Australia [A4study.org] (Sperling *et al*, 2014). All three of these trials are initially administering Aβ antibodies, but other agents targeting Aβ or other factors (tau; neuroinflammation) are planned or underway.

### Active vaccines: no longer at the forefront but not forgotten

AD immunotherapy in man began with an active vaccine trial (using AN‐1792, a synthetic Aβ1–42 peptide) that was terminated after 2–3 doses due to the occurrence of a T‐cell mediated meningeal inflammation in 6% of the Phase 2 patients (Gilman *et al*, 2005). But quantitative neuropathological analyses of a few brains from subjects who had died years after a Phase 1 trial of AN1792 revealed evidence of amyloid clearing and apparent lessening of neuritic dystrophy and synaptic deficits, compared to what would be expected in such advanced AD patients (Serrano‐Pozo *et al*, 2010). Only 1–2 trials of active Aβ vaccines are underway at this writing, but this approach clearly deserves more study, as the polyclonal antibody response may prove beneficial, and the cost and logistics of distributing passively administered monoclonal antibodies several times per year to the world's AD population are daunting.

### β–secretase 1 inhibition in Phase 3: much anticipated

Inhibitors of β‐secretase arrived later than those for γ‐secretase, in part because of the pharmacological challenges of targeting the large active site of this aspartyl protease in intact neurons. But now, several companies have Phase 2 or 3 trials of chemically distinct inhibitors underway. No published data of efficacy and side effects in man are yet available. Nevertheless, the discovery of many new substrates of β‐secretase, including some that are critical for signaling events in both the immature and mature nervous system (Willem *et al*, 2006; Hemming *et al*, 2009; Kuhn *et al*, 2012), raises the possibility of significant adverse events appearing over time in such trials. Speculation abounds about whether lowering Aβ42 monomers with β‐ or γ‐secretase inhibitors/modulators or binding and clearing plaques and diffusible Aβ with antibodies will turn out to be more efficacious. The combined testing of two anti‐Aβ agents is desirable and may not lie too far ahead.

## The amyloid hypothesis at 25 years

This review perforce mentions only a fraction of the many studies on the relationship of Aβ accumulation to the other features of the AD syndrome. But the examples we highlight underscore the compelling nature of the extensive preclinical and emerging clinical evidence that Aβ dyshomeostasis is upstream of alterations in other proteins and diverse cell types that contribute to the AD cognitive phenotype (Box 1). We emphasized more than a dozen years ago (Hardy & Selkoe, 2002) that definitive proof of this once controversial concept could only come from clinical trials that selectively target Aβ and produce slowing and ultimately arrest of cognitive decline in typical AD patients. The recent aducanumab Phase 1b data are consistent with such evidence, although we obviously need large, multi‐national trials that show significant amelioration of AD progression over 18–24 months.

Success breeds success, and it appears increasingly likely that exciting progress in the clinic, building upon a 3‐decade record of advances in the laboratory, will provide this proof. The continued push toward a safe and efficacious amyloid therapeutic takes nothing away from the need for alternative agents that target other early features of this complex and devastating syndrome. As others have pointed out (Small & Duff, 2008; De Strooper & Karran, 2016) and we concur, after disease initiation, the complexity of the downstream pathogenic processes increases. Nonetheless, it is likely that therapies aimed at these downstream processes will eventually have a role in the armamentarium against this devastating disease. It is not a question of one hypothesis against another. Rather, we must pursue multiple approaches, leading to a range of therapeutics that may together prevent the looming personal and societal tragedy that Alzheimer's disease has become.

## Conflict of interest

DS is a director of Prothena Biosciences and has received consulting fees from Janssen, Roche, and Sanofi and a speaker's fee from Biogen‐Idec. JH consults for Eisai and for Cytox and has received speaking fees from Eli Lilly, Takeda, and Roche.

**Pending issues**

What are the toxic species of Aβ and tau?

What is the connection between Aβ and tangle pathology? Is it direct and cell autonomous or does it involve non‐neuronal cells?

What is the mechanism of pathology spread and does understanding this spread provide therapeutic opportunities?

What is the function of APP and does Aβ have a function?

GWA studies have identified cholesterol metabolism, the innate immune system, and endosomal vesicle recycling as important pathogenic processes in AD: how do these relate to each other?
